# Anxiety and facial self-contacts: possible impact on COVID-19 transmission in dental practice

**DOI:** 10.1186/s12903-021-01564-6

**Published:** 2021-04-20

**Authors:** María Carrillo-Diaz, Laura Lacomba-Trejo, Antonio del Valle-González, Martín Romero-Maroto, María José González-Olmo

**Affiliations:** 1 Orthodontic and Pediatric Dentistry Department, Rey Juan Carlos University, Avda de Atenas s/n 28922, Alcorcón, Madrid, Spain; 2 Faculty of Psychology, Department of Personality, Assessment and Psychological Treatments, University of Valencia, Av. Blasco Ibáñez, 21, 46010 Valencia, Spain

**Keywords:** COVID-19, Touch, Dental fear, Anxiety, High risk

## Abstract

**Background:**

The purpose was to analyse the associations between dental and trait anxiety, fear of COVID-19 and the duration and frequency of spontaneous hand-to-face contact (self-contact).

**Methods:**

A cross-sectional design was carried out with 128 adult patients from four dental clinics in Madrid, during the confinement, from March 15 to May 15. The patients’ movements in the waiting room were monitored with Microsoft Kinect Software, also completed the Trait anxiety subscale of the STAI, the COVID-19 Fear and the S-DAI questionnaire.

**Results:**

Associations were observed between the duration and frequency of facial, mask and eye contact with trait anxiety and dental fear was determined only by the frequency of this self-contact. Trait anxiety is associated with dental anxiety and with fear of COVID-19. Although facial self-contact is higher in women, it also rises in men as dental fear increases. Moreover, dental anxiety is a good predictor of trait anxiety and the incidence of facial self-contact.

**Conclusions:**

Understanding the possible associations between biopsychosocial factors, such as trait anxiety, dental anxiety and self-contact is important. It may help to prevent the spread of COVID-19 in the population as well as enabling the formulation of effective interventions to improve oral health care through the implementation of dental care programmes.

**Supplementary Information:**

The online version contains supplementary material available at 10.1186/s12903-021-01564-6.

## Introduction

Starting in early 2020, a new human coronavirus, SARS-CoV-2 or the COVID-19 disease, became a global health problem, causing severe respiratory tract infections in humans and leading to a large number of deaths [1]. To date it is not known the pandemic’s overall impact on health systems and/or on dental services. In fact, during the first months of the pandemic, we did not have universal guidelines for providing dental care during the pandemic. As a result of not having an official protocol, the provision of dental care was completely halted or significantly reduced to only urgent dental care, which could have harmed patients’ oral health [2].

One of the main reasons for stopping dental care was that an asymptomatic person may be a potential source of transmission, so in dental clinics all patients must be treated as possible SARS-CoV-2 carriers [3]. In addition, COVID-19 can be transmitted from dry contaminated surfaces, including self-contamination via the mucous membranes of the nose, eyes or mouth [4]. Although literature on the transmission mechanisms of common respiratory infections is limited, contaminated hands in this context can spread respiratory infections [5].

Even though vaccines for SARS-COV-2 are being developed and approved, herd immunity or the accessibility of vaccines are not widely available to the public, so early containment and prevention of further spread are crucial in stopping the outbreak [6], therefore, preventing the virus’s spread in public and health-care settings is important [3].

Apart from physical health concerns, the COVID-19 pandemic also poses psychosocial consequences, such as: fear of self- contamination or spreading the contagion to people close to them; stress from continuous economic, political and social changes; emotional problems resulting from isolation; and distress from the possibility or reality of losing a loved one [7]. All these circumstances exacerbate patients’ premorbid profiles (socioeconomic difficulties, personality, physical and mental health problems, etc.) [8]. If we consider differences associated with gender, studies consistently show that females have more anxiety [9]. Pre-pandemic factors also should be considered; the literature has shown that patients commonly experience stress when undergoing dental treatment. In fact, the prevalence of dental fear has reached 24.3%, a figure which has been maintained over the last decade among the adult population [10]. In addition, patients with greater neuroticism, such as high trait anxiety or other anxiety disorders, are more likely to fear dental care [11, 12]. These emotions are associated with avoidance of dental care, which can negatively impact the course, treatment, and outcome of dental pathologies [13].

According to Lang’s (1968) theory of the three response systems, anxiety manifests via a triple-response system [14] that includes cognitive, behavioural, and physiological aspects [15]. In this sense, the varied psychophysiological responses to acute stress may include: tremors, tachycardia, sweating, and increased movements, such as touching the face and/or manipulating objects [16]. These movements occur unconsciously and can help regulate emotions [17]. It has been observed that people with anxiety or dental anxiety present a higher heart rate, higher heart rate variability and lower skin conductivity [18], as well as a greater amount of movement [15], but the association of these factors with facial self-contact has not been studied. This aspect is especially interesting because if people can touch their faces approximately 30 times an hour in situations that do not elicit anxiety [19], the number of facial self-contacts may increase in stressful situations [17] and increased facial self-contact may help transmit and spread the SARS-CoV-2 [20].

Therefore, the aim of the present research was to analyse the relationship between dental fear, trait anxiety, fear of COVID-19 and duration and frequency of facial self-contact; confirm gender differences in trait anxiety, dental anxiety and fear of COVID-19; and assess gender differences in facial self-contact related to dental anxiety, as well as to determine if dental anxiety is a mediating factor between trait anxiety and frequency of facial self-contact.

## Method

### Design type

This observational, descriptive and cross-sectional design research was carried out in Spain from March 18 to May 15, 2020, during the initial stage of the pandemic. Since the World Health Organization officially declared the global pandemic, Madrid was established as the epicenter of COVID-19 cases in Europe. By May 15, in Madrid, there were 69,734 infected cases recorded, 38,456 hospitalized patients and 8,723 deaths.

### Data collection

The participants comprised 124 adult patients (over 18 years old), who had not suffered from COVID-19 and who visited one of four dental clinics in Madrid. Four orthodontic dental clinics were randomly selected from the 10 dental clinics that were then open and willing to participate with the research team. At the time of data collection, dental activity in Spain was limited exclusively to emergency treatment by the Spanish Order SND/310/2020 issued on March 31. All adult patients who called these dental clinics for an appointment to resolve an orthodontic emergency in the selected time period were invited to participate. A total of 150 subjects called, of which 26 (17.3%) refused to participate. The patients selected were regular orthodontic patients of the dental clinics and knew the clinic staff. The study’s objectives and nature were explained beforehand by telephone, and patients who agreed to participate in the study were enrolled. At this point, patients were informed that this appointment would be exclusively for diagnosis and another appointment for treatment would be scheduled.

Patients were asked to come to the dental clinic at the agreed-upon time (to avoid unnecessary waiting) unaccompanied and with a mask. One patient was scheduled per hour to avoid person-to-person contact. Upon arriving at the clinic, patients were asked to rub their hands with a hydroalcoholic gel and to put on shoe covers, as established by the protocol for preventing COVID-19 transmission in Spain [21].

The waiting room was empty, except for the participant and the same member of the research team, who explained the process. The patients were informed that their behaviour in the waiting room would be observed for a study. To blind them to the study, they were not informed about which behaviours were under observation to minimize the potential for behavioural changes due to being observed [22]. After entering the waiting room, participants signed the informed consent form before their movements were monitored and were instructed to sit in a chair. The mask remained over the mouth and nose the whole time in the waiting room. The movement monitoring time was the minimum time for preparation and disinfection of the dental chair area. The average time in the waiting room was 10.2 (± 3.8) minutes. The amount and duration of facial self-contact were independently monitored, as were the number and duration of contacts made to the mask and eyes.

After this recording, participants filled out self-administered instruments using Google Forms to avoid further contact. The questionnaire’s link was sent via e-mail or WhatsApp to their mobile device, and they completed it before entering the dental office.

This research is approved by the King Juan Carlos University Ethics and Research Committee.

### Instruments

An ad hoc questionnaire was developed to evaluate socio-demographic variables of age, gender, and educational level.

The *Microsoft Kinect* was used to evaluate the detection and counting of movement patterns [23].

Anxiety symptomatology was evaluated as a trait using the trait anxiety subscale of the *State–Trait Anxiety Inventory* (STAI) [24]. Fear of COVID-19 was assessed using the *COVID-19 Fear Questionnaire* (FCV-19S) developed by Ahorsu and colleagues (March, 2020) [25].

The short version of the *Dental Anxiety Inventory* (S-DAI) [26] was used to assess the physical reactions, thoughts, and behavioural aspects of people’s dental fear. The description of the instruments is attached in the Additional file 1: online Appendix.

### Statistical analysis

The study presents a cross-sectional study considering the variables described in the previous section. A statistical analysis was performed using SPSS version 26 (SPSS Inc., Chicago, IL, USA). The data analysis included descriptive statistics, the Kolmogorov–Smirnov test to evaluate the assumption of normality, which was confirmed (*p* > 0.05 in all comparisons). In addition, analysis of differences in means with t-test for which the effect size was calculated using Cohen’s *d*. A low effect size (ES) was considered *d* ≈ 0.2, medium was ≈ 0.5, and high was ≈ 0.8 [27]. The relationships between variables were analyzed using Pearson’s correlations. Significance levels were established at 0.05. Cronbach's alpha was also carried out to evaluate the internal consistency of the instruments.

**Fig. 1:**
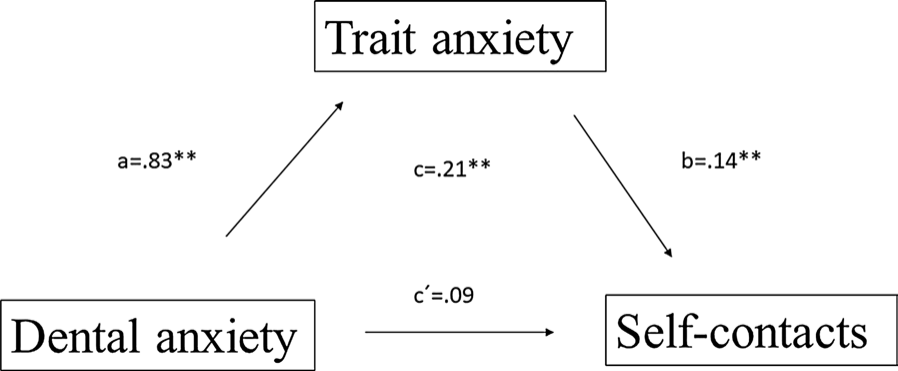
Mediational anxiety analysis feature in the association between dental anxiety and facial self-contacts. *Note* A = Effect of independent variable on mediating variable. B = Effect of mediating variable on dependent variable. C = direct effect. C’ = indirect effect. ** = Significant at the 0.01 level

Subsequently, a PROCESS module (version 3.3) by Hayes [28] for SPSS, was used to perform moderation models (model 1—Table 3) to observe if sex moderates the relationship between dental anxiety and self-contacts and multiple simple mediation analyses (model 4—Table 4 and Fig. 1) to observe whether trait anxiety is a mediating factor between dental anxiety and facial self-contacts.

## Results

The sample was comprised of 64 (51.6%) men and 60 (48.4%) women, with an age range of 24 to 62 years (41.2 ± 11.4). In terms of educational levels for the total sample, 55.6% had completed primary school, 22.6% had completed secondary school, and 21.8% had obtained a university degree.

Facial self-contact patterns were observed in 93.5% of the registered patients, with an average of 7.1 ± 4.8 contacts, 4.1 ± 2.9 for mask contacts and 3 ± 1.9 for eye contacts. The average time in the waiting room was 10.2 ± 3.8 min, with 0.7 ± 0.6 facial self-contacts per minute, 0.4 ± 0.4 mask contacts per minute and 0.3 ± 0.2 eye contacts per minute registered. The average duration of facial contact was 0.6 ± 0.2 contacts per minute, with 0.3 ± 0.1 contacts per minute for the mask and 0.2 ± 0.1 contacts per minute for the eyes. The median and *SD* score found for trait anxiety (22.6 ± 18.1), fear of COVID-19 (17.4 ± 9.1) and dental anxiety (19.1 ± 8.3).

Table 1 reveals statistically significant associations between the number and duration of facial, mask and eye self-contact with trait anxiety and dental fear. Similarly, trait anxiety was associated with higher dental fear in general (*r*^2^ = 0.261**) and with fear of COVID-19 (*r*^2^ = 0.273**). Dental fear was associated with the frequency in face (*r*^2^ = 0.194*), mask (*r*^2^ = 0.196*) and eyes (*r*^2^ = 0.200*) contacts but not the duration of contact. The association of self-contacts with trait anxiety was moderate to high, but fear of COVID-19 generally showed weak linear associations.

**Table 1:** Pearson correlations coefficients (*r*^2^) for the variables studied (Trait anxiety, Dental fear, Fear of COVID-19, Frequency and duration of self-contact) *n* = 124

	2	3	4	5	6	7	8	9
Anxiety-trait	0.261**	0.273**	0.620**	0.620**	0.617**	0.706**	0.692**	0.674**
Dental fear		0.410**	0.194*	0.196*	0.200*	0.167	0.159	0.158
Fear of COVID-19			0.114	0.098	0.128	0.111	0.090	0.129
Face contact frequency				0.993**	0.986**	0.923**	0.923**	0.881**
Mask contact frequency					0.966**	0.918**	0.933**	0.850**
Eyes contact frequency						0.909**	0.884**	0.905**
Duration of face contact							0.986**	0.965**
Duration of mask contact								0.911**
Duration of eye contact								

*Correlation is significant at the 0.05 level

**Correlation is significant at the 0.01 level

We examined mean differences in sociodemographic factors (age, gender and educational level) regarding self-contacts. Statistically significant differences were only found concerning participants’ gender. As shown in Table 2, women showed more frequent self- contact of the face in general (*p* < 0.05), and of their eyes and mask in particular (*p* < 0.05). They had longer facial and eye contact (*p* < 0.05), greater trait anxiety (*p* < 0.01) and greater dental fear (*p* < 0.01). Fear of COVID-19 was observed as having a small effect, while the extent of trait anxiety, frequency and duration of self-contacts was assessed as medium and dental fear, large.

**Table 2:** Differences in gender for trait anxiety, fear of COVID-19, dental fear, frequency and duration of self-contact

		Gender				
	Mean (*SD*)	Mean (*SD*)	Mean (*SD*)	*t*	*p*	*d*
	Sample	Man	Woman			
	*n* = 124	*n* = 64	*n* = 60			
Trait-anxiety	22.6 (18.1)	17.7 (15.7)	27.7 (19.1)	3.15	0.002**	0.56
Dental-fear	19.1 (8.3)	16 (6.7)	22.3 (8.7)	4.46	0.001**	0.80
Fear of COVID-19	17.4 (9.1)	16.3 (8.8)	18.5 (9.2)	1.38	0.171	0.24
Face contact frequency	7.1 (4.8)	6.2 (4.2)	8.2 (5.2)	2.28	0.024*	0.42
Mask contact frequency	4.1 (2.9)	3.6 (2.6)	4.8 (3.1)	2.25	0.026*	0.41
Eyes contact frequency	3 (1.9)	2.6 (1.6)	3.4 (2.1)	2.34	0.021*	0.42
Duration of face contact	0.7 (0.6)	0.5 (0.2)	0.6 (0.3)	2.01	0.047*	0.37
Duration of mask contact	0.4 (0.4)	0.3 (0.1)	0.3 (0.1)	1.97	0.051	0.33
Duration of eye contact	0.2 (0.1)	0.2 (0.1)	0.2 (0.1)	2.00	0.048*	0.34

*Significant at the 0.05 level

**Significant at the 0.01 level

*SD* = Standard deviation*. d* de Cohen; Effect size small ≈ 0.20; Effect size medium ≈ 0.50; Effect size large ≈ 0.80

The moderating role of gender was evaluated (Table 3). The interaction between gender and dental anxiety significantly increased the coefficient of determination (*F* = 3.93; ∆R2 = 0.09; *p* ≤ 0.05). With regard to the conditional effects, the impact of gender on self- contact was significant for men (*t* = 2.53; *p* ≤ 0.05; 95% CI = [0.05, 0.39]), but not for women (*t* =  − 0.14; *p* > 0.05; 95% CI = [− 0.15, 0.13]). Thus, we observed that the amount of self-contact for women is independent of dental anxiety. However, in the case of men, as dental fear increases, so does facial self-contact.

**Table 3:** Moderating effects of sex on the relationship between trait anxiety and self-contacts

	Effect	*SE*	*t*	*p*	LLCI	ULCI
*Model* *R*^*2*^ = .09*; F* = *3.93; p* ≤ *.05*						
Dental anxiety	0.22	0.09	2.53	0.01	0.05	0.39
Sex	5.68	2.24	2.53	0.01	1.23	10.12
Dental anxiety * sex	− 0.23	0.12	− 2.06	0.04	− 0.45	− 0.01
*Conditional effects*						
Men	0.22	0.09	2.53	0.01	0.05	0.39
Woman	− 0.01	0.97	− 0.14	0.89	− 0.15	0.13

Bootstrap samples = 10,000. *R*^2^ = Coefficient of determination. *SE* = Standard error. LLCI = Lower level of the 95% confidence interval. ULCI = Upper level of the 95% confidence interval

We explored the possibility that dental anxiety could be a moderating factor between trait anxiety and the frequency of facial self-contact. With respect to the moderation analysis, no significant interaction effects were observed between trait anxiety and facial self-contact, considering dental anxiety as a moderator (*t* = 2.39; *p* ≤ 0.05; 95% CI = [0, 0.01]). Finally, to determine the trait anxiety’s mediating capacity between dental anxiety and regression data, dental anxiety is a good predictor of trait anxiety (*β* = 0.83, *p* < 0.01) and of the number of facial self-contacts (*β* = 0.14; *p* < 0.01). In addition, trait anxiety was directly associated with the number of facial self-contacts (*β* = 0.09, *p* < 0.01). Indirect relationships, which result from including dental anxiety and trait-anxiety in a multiple regression equation with the number of self-contacts as the criterion, showed trait anxiety as a statistically significant mediator between dental anxiety and the number of facial self- contacts (*β* = 0.12, *p* < 0.01) (Table 4).

**Table 4:** Mediational anxiety analysis feature in the association between dental anxiety and facial self-contacts

IV	M	DV	R^2^	*p*	Efect of IV on M	Effect of M on DV	Direct effect	Interdirect effect	96% CI for indirect effects	Total effect
Dental anxiety	Trait-anxiety	Self-contacts	0.21	0.001	0.83**	0.09*	0.14**	0.12**	0.07–to 0.17	0.21**

Bootstrap samples = 10,000. *R*^2^ = Coefficient of determination

*Significant at the 0.05 level. **Significant at the 0.01 level

Therefore, more dental anxiety increases the number of facial self-contacts, but this is because greater dental anxiety results from greater trait anxiety, which therefore yields more self-contacts (Fig. 1).

## Discussion

The COVID-19 pandemic has presented psychological consequences and the dental surgery is no exception. This study makes innovative contributions regarding the association of facial self-contact frequency and duration in dental clinic patients with psychosocial factors such as dental fear, fear of COVID-19 and trait anxiety, because such self-contact could have an impact on the transmission of SARS-CoV-2.

Our data partially align with previous studies, such that trait anxiety and dental fear relate to a subject’s amount of facial self-contact, but fear of COVID-19 does not seem to relate [15, 18]. However, COVID-19-related fear is very specific and related to (possible or perceived risk of) contamination. This perception would be associated with a hypervigilance against threats (i.e., “germs”) and hygienic behaviours such as avoiding facial self-contact [29]. Previous research has pointed out the association between anxiety and frequency of facial self-contact [17, 30]. However, no study has analysed the relationship between fear of COVID-19 and facial self-contact, an issue we address in our research to make an interesting contribution to this current scientific problem.

Our study is unique, too, in its investigation of the gender variable moderation in the relationship between dental anxiety and facial self-contact, as well as the trait anxiety variable mediation between dental fear and facial self-contact. We also aimed to understand how facial self-contact frequency might be associated with dental anxiety and fear of COVID-19.

Our data show that dental anxiety and trait anxiety are higher in women, as others have previously reported [31, 32]. We also show that the frequency of self-contact is higher in women, but according to the moderation analyses, the number of self-contacts increased in men as dental anxiety increased, but this relationship did not occur for women. As the literature consistently points out, women tend to show more anxiety in general, it seems that in the case of men there may be a greater physiological and motor expression, although these data are still contradictory [31]. We observed that self-contact in women were independent of the dental anxiety they presented; we therefore point out that this aspect needs to be studied in depth in future research.

In reference to facial self-contacts, we have to clarify that our participants all wore masks; however, the frequency of self-contact was similar to that reported without masks [19]. On the other hand, trait anxiety was a mediating factor for the number of facial self-contacts in the waiting room when subjects faced dental fear. This implies that, even with similar levels of dental fear, people with less trait anxiety will make fewer facial self-contacts to the eyes and mask and therefore could be more protected from infection. These data provide new insights into who may be at greater risk for contracting COVID-19, since numerous studies have linked touching a mask by hand with an increased risk of infection and cross-contamination [33, 34].

Despite our study’s contributions, the results should be interpreted with caution. First, the study sample was small because the participants were recruited during Spain’s period of confinement and were patients who attended the clinics due to an emergency. In future studies the sample should be larger to increase the statistical power of the study. In addition, we used convenience sampling, so subsequent studies are necessary to continue investigating the reality of the patients who attend dental clinics via probability sampling. Future studies should monitor the time each patient spends in the waiting room, the number of times patients wash their hands, and touch surfaces, as these may be associated with a greater likelihood of infection. Furthermore, thoughts associated with anxiety could be evaluated qualitatively, and other behaviours or physiological responses associated with anxiety, such as heart rate or skin conductance, could be evaluated quantitatively [35].

In addition, using questionnaires can lead to social desirability bias, but objective evaluation methodologies significantly reduce the impact of this bias. In our research, both measures were consistently associated. Importantly, the above results do not indicate causal relationships between the variables of interest. The present investigation’s design only allows us to conclude that significant relationships exist between the variables. Thus, more ample future studies are required to clarify the obtained results and to evaluate the temporary reliability of our data.

Two groups of applications for dentistry are obtained from our results, depending on whether the practical implications are considered via the prevention of COVID-19 or via psychological intervention.

From the point of view of COVID-19 prevention:

The WHO recommends “ensuring that environmental cleaning, and disinfection procedures are followed consistently and correctly.” SARS-CoV-2 can persist on inanimate surfaces for a long time, but can be effectively inactivated via surface disinfection in 1 min using 62–71% ethanol, 0.5% hydrogen peroxide, or 0.1% sodium hypochlorite [6]. Every time a patient leaves the waiting room, their chair, and environment must be disinfected before another patient takes that place to avoid cross-contamination.

From the point of view of psychological intervention:

Based on the results obtained, we propose the inclusion of psychology professionals in the circuit of evaluation and intervention of patients who come to dental clinics. A psychological evaluation prior to the dentistry consultation could help to detect the patients who need a previous intervention. In this way, health psychologists could attend to patients who require this with psychoeducation, relaxation techniques, rational- emotive therapy or the use of virtual reality [36, 37], so that when they arrive at the consultation, their level of discomfort is lower, and therefore facial self-contact will be reduced. These techniques, among others, could be applied in an online group format where patients could increase their level of social support, but the chances of contagion would be reduced. Other procedures could include caring for the work environment, such as using music therapy to establish a calming atmosphere in the waiting room and treatment rooms [38].

Future research should evaluate the psychological and medical aspects, considering an integral and multidisciplinary approach to health from a biopsychosocial point of view.

## Conclusions

This study presents important contributions to clinical practice in dentistry during the COVID-19 pandemic. It shows how the behaviour of people in the waiting room of the dental clinic may be influenced by psychological and sociodemographic factors, and this could contribute to the spread of COVID-19. In particular, we observe that there is a positive association between the number of facial self-contacts with trait anxiety and dental anxiety. However, there is no association between self-contacts and fear of COVID-19. For their part women make a greater number of facial self-contact and have higher levels of trait and dental anxiety. These gender differences in self-contact do not occur with respect to fear of COVID-19. On the other hand, the relationship between dental anxiety and self-contact was moderated by gender in the case of men. In men, the greater the dental anxiety, the greater the amount of facial self-contact. Finally, the relationship between dental anxiety and self-contacts is mediated by trait anxiety. This would point to anxious symptoms, fear and socio-demographic factors as potentially influential in the presence of a greater number of facial self-contacts, and with it, their possible association with the spread of the virus.

## Supplementary Information

**Additional file 1:** Additional information about the instruments used.

## Data Availability

All of the material is owned by the authors and/or no permissions are required. The datasets generated during and analyzed during the current study are not publicly available due to [national data protection law] but are available from the corresponding author on reasonable request.
