# Supplementary material for: Anxiety and facial self-contacts: possible impact on COVID-19 transmission in dental practice
Source: BMC Oral Health. 2021 Apr 20;21:200. doi: 10.1186/s12903-021-01564-6 (PMC8056369; doi:10.1186/s12903-021-01564-6)
Supplement: Supplementary file 1 — Additional file 1: Additional information about the instruments used. [file 12903_2021_1564_MOESM1_ESM.docx]

***Online Appendix***

***Instruments:***

The *Microsoft Kinect* was used to evaluate the detection and counting of movement patterns. This system was originally designed to locate movement for video game controls, but it has been used effectively in numerous health studies, including to assess stress patterns [1], to monitor breath for breast cancer detection [2] or to monitor the movement of people with Parkinson's disease [3]. In addition, previous studies have confirmed the reliability of kinematic measurements using Microsoft Kinect, even during walking or active movement [4].

Microsoft Kinect’s usefulness has been endorsed in numerous movement recognition studies concerning postural control [5], neck angle [6] and spatial-temporal aspects of steps [7]. Microsoft Kinect comprises an RGB camera, a depth-detection system, and infrared and CMOS (complementary metal-oxide semiconductor) sensors, which allow mapping images and recognizing movements in 3 dimensions. Each pixel in the depth image is assigned a vector value (X, Y, D) where X and Y represent the coordinate values, and D represents the distance between the pixel and the sensor.

Microsoft Kinect can generate a virtual skeleton by locating and recording the movement of joints over time. It also has a facial recognition system to identify various key points on the head. In this case, it was configured to record the touches the patients made to their mask and eyes (considering the sum of both to be the number of facial contacts). The time in seconds of each contact and the time the patient spent in the waiting room were also monitored.

Anxiety symptomatology was evaluated as a trait using the trait anxiety subscale of the *State–Trait Anxiety Inventory* (STAI). The STAI is a self-report questionnaire comprising a state-anxiety subscale (how one feels in a particular time or situation) and a trait-anxiety subscale (a relatively stable anxious propensity that characterizes individuals who tend to perceive situations as threatening) [8]. STAI has been successfully employed in health research and is widely used in the dental field [9, 10]. The State-Trait Anxiety Inventory comprises 20 items answered using a Likert-type scale ranging from 0 (almost never) to 3 (almost always). Representative items include statements such as “I feel nervous” and “I feel worried.” The total score is obtained by adding up the items while considering the inverted reverse-scored items. The psychometric properties of the STAI are satisfactory [11, 12]. The internal consistency of the STAI in the present study was very good (α = .94).

Fear of COVID-19 was assessed using the *COVID-19 Fear Questionnaire* (FCV-19S), recently developed by Ahorsu and colleagues (March, 2020) [13]. The scale has 7 items answered using a Likert-type scale ranging from 1 (strongly disagree) to 5 (strongly agree). Example of an item: “I am most afraid of coronavirus”, The total score is obtained by summing the items (scores range from 7 to 35), and higher scores indicate a greater fear of COVID-19. The psychometric properties of the FCV-19S have been found satisfactory in prior research [14]. The internal consistency of the FCV-19S in the present study was very good (α = .86).

The short version of the *Dental Anxiety Inventory* (DAI) [15] was used to assess the physical reactions, thoughts, and behavioural aspects of people’s dental fear. This version has 9 items that are answered from 1 (totally untrue) to 5 (completely true), and higher scores indicate greater dental anxiety. Example of an item: “I become nervous when the dentist invites me to sit down in the chair”. The total score is obtained by adding up the items. Each subscale has 3 items with scores ranging from 3 to 15. The psychometric properties of the DAI have been found satisfactory in prior research [16]. The internal consistency of the DAI in the present study was very good (α = .882).

**References**

1. Giakoumis D, Drosou A, Cipresso P, Tzovaras D, Hassapis G, Gaggioli A, et al. Real- time monitoring of behavioural parameters related to psychological stress. Stud health technol inform. 2012;181:287-91.
2. Edmunds DM, Gothard L, Khabra K, Kirby A, Madhale P, McNair H, et al. Low-cost Kinect Version 2 imaging system for breath hold monitoring and gating: Proof of concept study for breast cancer VMAT radiotherapy. J Appl Clin Med Phys. 2018;19:71-8. https://doi.org/10.1002/acm2.12286
3. Galna B, Barry G, Jackson D, Mhiripiri D, Olivier P, Rochester L. Accuracy of the Microsoft Kinect sensor for measuring movement in people with Parkinson’s disease. Gait Posture. 2014;39:1062-8. https://doi.org/10.1016/j.gaitpost.2014.01.008
4. Tamura H, Tanaka R, Kawanishi H. Reliability of a markerless motion capture system to measure the trunk, hip and knee angle during walking on a flatland and a treadmill. J Biomech. 2020;109:109929. https://doi.org/10.1016/j.jbiomech.2020.109929
5. Clark RA, Pua Y-H, Fortin K, Ritchie C, Webster KE, Denehy L, et al. Validity of the Microsoft Kinect for assessment of postural control. Gait Posture. 2012;36:372–7. https://doi.org/10.1016/j.gaitpost.2012.03.033
6. Allahyari T, Sahraneshin Samani A, Khalkhali H-R. Validity of the Microsoft Kinect for measurement of neck angle: comparison with electrogoniometry. Int J Occup Saf Ergon. 2016;23:524–532. https://doi.org/10.1080/10803548.2016.1219148
7. Springer S, Yogev Seligmann G. Validity of the Kinect for Gait Assessment: A Focused Review. Sensors. 2016;16:194. https://doi.org/10.3390/s16020194
8. Spielberger CD, Gorsuch RL, Lushene R, Vagg PR, Jacobs GA. Manual for the State- Trait Anxiety Inventory. Palo Alto, CA: Consulting Psychologists Press;1983.
9. Peñacoba C, González MJ, Santos N, Romero M. Psychosocial predictors of affect in adult patients undergoing orthodontic treatment. Eur J Orthod. 2014;36:93-8. https://doi.org/10.1093/ejo/cjt007
10. Romero-Maroto M, Santos-Puerta N, González-Olmo MJ, Peñacoba-Puente C. The impact of dental appearance and anxiety on self-esteem in adult orthodontic patients. Orthod Craniofac Res. 2015;18:143-55. https://doi.org/10.1111/ocr.12091
11. del Río Olvera FJ, Santamaría FC, Cabello García MA, Vela JA. State Trait Anxiety Inventory (STAI): Psychometric analysis and functioning in a sample of drug addicts and controls. Univ Psych. 2018;17:1-10. <http://dx.doi.org/10.11144/javeriana.upsy17-1.caer>
12. Charles D, Speilberger P, Vagg R. Psychometric Properties of the STAI: A Reply to Ramanaiah, Franzen, and Schill. J Pers Asses. 1984;48: 95-7. https://doi.org/10.1207/s15327752jpa4801_16
13. Ahorsu DK, Lin CY, Imani V, Saffari M, Griffiths MD, Pakpour AH. The Fear of COVID-19 Scale: Development and Initial Validation. Int J Ment Health Addict. 2020;27:1-9. https://doi.org/10.1007/s11469-020-00270-8
14. Martínez-Lorca M, Martínez-Lorca A, Criado-Álvarez JJ, Cabañas-Armesilla MD, Latorre JM. The fear of COVID-19 scale: Validation in spanish university students. Psychiatry Res. 2020;2;293:113350. https://doi.org/10.1016/j.psychres.2020.113350
15. Liu Y, Huang X, Yan Y, Lin H, Zhang J, Xuan D. Dental fear and its possible relationship with periodontal status in Chinese adults: a preliminary study. BMC Oral Health. 2015; 28;15-8. https://doi.org/10.1186/1472-6831-15-18
16. Aartman IH. Reliability and validity of the short version of the Dental Anxiety Inventory. Community Dent Oral Epidemiol. 1998;26:350-4. https://doi.org/10.1111/j.1600-0528.1998.tb01972.x
